# Endothelial activation in thromboangiitis obliterans: mechanisms and therapeutic horizons

**DOI:** 10.3389/fimmu.2025.1668203

**Published:** 2025-08-27

**Authors:** Wanting Wang, Siyao Chang, Gang Zhao

**Affiliations:** ^1^ Heilongjiang University of Chinese Medicine, Heilongjiang, Harbin, China; ^2^ Vascular Surgery Department, Harbin Fifth Hospital, Harbin, China; ^3^ Department of Peripheral Vascular Disease, First Affiliated Hospital, Heilongjiang University of Chinese Medicine, Harbin, China

**Keywords:** thromboangiitis obliterans, endothelial cell, immune dysregulation, mitochondrial dysfunction, copper/iron homeostasis, thrombogenicity, therapeutic interventions

## Abstract

Thromboangiitis obliterans (TAO) is a non-atherosclerotic, inflammatory vasculopathy characterized by thrombotic occlusion of small- and medium-sized vessels, leading to tissue ischemia and gangrene. Emerging evidence underscores endothelial cell (EC) activation as a central driver of disease progression, mediated by immune dysregulation, oxidative stress (Nrf2/ROS imbalance), impaired nitric oxide signaling (eNOS/iNOS dysregulation), endoplasmic reticulum and mitochondrial dysfunction, and disrupted copper/iron homeostasis. These pathways collectively promote a prothrombotic, proinflammatory endothelial phenotype, perpetuating vascular injury. Current therapies primarily alleviate symptoms but fail to address underlying EC dysfunction. Recent advances, including stem cell therapy and targeted immunomodulation, offer promising avenues for restoring endothelial homeostasis. However, translating mechanistic insights into durable clinical benefits requires further research into precision medicine approaches and large-scale validation of novel therapeutics. This review summarizes the multifactorial pathogenesis of TAO, emphasizing EC activation as a therapeutic linchpin, and outlines future directions to bridge translational gaps in disease management.

## 1 Introduction

Thromboangiitis obliterans (TAO) is a chronic, non-atherosclerotic, segmental vasculitis characterized by inflammatory thrombi affecting small- and medium-sized arteries and veins of the extremities, often progressing to ulcers or gangrene (1). Arterial insufficiency leads to claudication, Raynaud’s phenomenon, and, in severe cases, amputation. The distal distribution and poor collateral circulation render surgical and endovascular approaches largely ineffective. Although the precise pathogenesis remains unclear, accumulating evidence suggests an autoimmune component, with various autoantibodies and immune cells targeting vascular structures (2–4). Histologically, TAO features a cellular thrombus rich in polymorphonuclear leukocytes, mononuclear cells, and giant cells, with minimal vessel wall involvement (5). Conventional inflammatory markers (ESR, CRP) and common autoantibodies may remain normal during acute episodes, yet immune dysregulation is believed to drive disease activity (6). Autoimmune features are frequently observed, including elevated anti-endothelial cell antibodies (AECAs), especially during active disease. These antibodies bind not only to surface but also intracellular endothelial antigens, implicating endothelial dysfunction in disease progression (7).

Endothelial dysfunction in TAO is often preceded by endothelial activation, marked by increased expression of adhesion molecules such as ICAM-1 and VCAM-1, which promote leukocyte adhesion and a prothrombotic vascular phenotype. Elevated circulating ICAM-1 levels further support this persistent endothelial activation (8). This process is primarily mediated by pro-inflammatory cytokines—particularly TNF and IL-6—which enhance leukocyte recruitment and adherence to the endothelium (9). Thus, endothelial activation constitutes a central mechanism in TAO pathophysiology and represents a potential therapeutic target. This review highlights recent insights into the role of endothelial cell activation in TAO and its implications for future therapeutic interventions.

## 2 Mechanisms of endothelial cell activation in TAO

### 2.1 Immune complex-mediated endothelial activation

Endothelial cell (EC) activation can be broadly classified into two types: Type I activation, a rapid response independent of new gene expression (also termed stimulation), and Type II activation, a slower but sustained response requiring *de novo* gene transcription. Acute inflammation involves the rapid recruitment of neutrophils within hours, driven by EC activation—a process by which resting endothelial cells acquire new functional properties (10). When acute stimuli persist, particularly under sustained adaptive immune responses, inflammation transitions toward chronicity. Type I activation is typically initiated by heterotrimeric G protein–coupled receptor ligands, whereas Type II activation is driven by proinflammatory cytokines such as tumor necrosis factor (TNF) and interleukin-1 (IL-1), involving monocytes, effector, and memory T cells. Adaptive cytokines, including interferon-γ (IFN-γ) and IL-4, further reprogram Type II–activated ECs, reinforcing T-helper cell polarization and amplifying inflammation (11). In patients with TAO, high titers of biologically active circulating mixed immune complexes have been observed, suggesting a pivotal role for immune complex–induced EC Type II activation and neutrophil-mediated endothelial injury in TAO pathogenesis (12).

### 2.2 Involvement of immune and inflammatory cytokines in endothelial activation

Given their anatomical interface with the circulation, endothelial cells (ECs) are persistently exposed to immune mediators and represent principal targets of pro- and anti-inflammatory cytokines. In TAO, heightened inflammation activates ECs via TNF-α, IL-6, and IL-17, thereby enhancing leukocyte adhesion and transmigration (9). Histological evidence reveals abundant CD4^+^ T cells in the intima and near thrombi, with macrophage infiltration observed in early disease stages (2). Differentiation of CD4^+^ T cells into Th1, Th2, Th17, and Treg subsets orchestrates divergent immune programs: Th1/Th17 cells amplify inflammation through IFN-γ and IL-17, whereas Th2/Treg cells secrete IL-4 and IL-10 to mediate immunoregulation. Elevated IFN-γ, IL-17, and TNF-α levels in TAO patients correlate strongly with EC activation (13). HMGB1 is markedly upregulated in TAO patient plasma, promoting inflammation and EC dysfunction by enhancing ICAM-1 expression and cytokine release (14). Moreover, immunoadsorption-based antibody removal has shown potential in attenuating disease severity. While associations with MHC class I and II alleles have been reported, genetic testing remains absent from clinical application (5).

Recent studies underscore the role of IL-33 in TAO. Endothelial cells, as IL-33 targets, respond via steroid-resistant proinflammatory signaling pathways. IL-33 binds the transmembrane receptor ST2L and recruits the adaptor MyD88, which in turn engages IRAK1/IRAK4 and TRAF6 to activate the canonical NF-κB pathway and the MAPK modules p38, JNK, and ERK (15–18), In endothelial cells, this signaling increases transcription of adhesion molecules (ICAM-1, VCAM-1, E-selectin), chemokines (CXCL1, CCL2), and prothrombotic mediators (tissue factor, PAI-1), thereby amplifying leukocyte tethering, transmigration, and thrombogenicity (19–22). Notably, IL-33–driven gene programs show relative steroid resistance, glucocorticoids incompletely suppress NF-κB/MAPK outputs downstream of MyD88/IRAK/TRAF6, providing a plausible explanation for the limited efficacy of steroid-based regimens in subsets of TAO patients with elevated circulating IL-33 (23).

### 2.3 Oxidative stress and endothelial activation

Excessive oxidative stress elevates intracellular reactive oxygen species (ROS) in endothelial cells, upregulating adhesion molecules including MCP-1, ICAM-1, VCAM-1, and E-selectin (24), thereby facilitating monocyte adhesion, endothelial activation, and tissue infiltration (25). Nuclear factor erythroid 2-related factor 2 (Nrf2), a member of the Cap’n’collar basic-region leucine zipper (CNC-bZIP) transcription factor family, serves as a master regulator of the cellular antioxidant response. Under oxidative or pathological stimuli, phosphorylated Nrf2 dissociates from its cytoplasmic repressor and translocates into the nucleus, where it induces the expression of downstream genes encoding antioxidant enzymes, such as heme oxygenase-1, superoxide dismutase, and glutathione peroxidase. This cascade facilitates the detoxification of reactive oxygen species (ROS) and confers cytoprotection by mitigating oxidative stress, inflammation, and apoptosis (26). This program detoxifies ROS and mitigates oxidative stress–driven inflammation and apoptosis. Impaired Nrf2 signaling blunts these defenses, enabling ROS accumulation, mitochondrial injury, cytochrome c release, and caspase activation, thereby predisposing endothelial cells to apoptosis (27). Concurrently, sustained oxidative stress under Nrf2 deficiency augments NF-κB activity, transcriptionally upregulating ICAM-1 and VCAM-1, which amplifies leukocyte recruitment and sustains vascular inflammation (28–31). In TAO, chronic arterial occlusion and hypoxia exacerbate oxidative burden, further mismatching oxygen supply and demand (32, 33). This imbalance contributes to cellular dysfunction or death. Therefore, therapeutic strategies targeting oxidative stress and modulating Nrf2-related signaling pathways represent promising approaches for ameliorating vascular inflammation in TAO (34, 35).

### 2.4 Inducible nitric oxide synthase mediates endothelial cell activation

Vascular endothelial cells form a specialized barrier between blood and vessel walls, regulating vascular tone, blood pressure, coagulation balance, and vascular permeability to maintain homeostasis (10). Hemodynamic forces, particularly shear stress, play a central role in endothelial integrity. In TAO, shear stress-induced activation of endothelial nitric oxide synthase (eNOS) and nitric oxide (NO) production is impaired, compromising vasodilation (36). Pathological stimuli reduce eNOS expression and NO bioavailability while upregulating hypoxia-inducible factor-1 (HIF-1), endothelin-1 (ET-1), and plasminogen activator inhibitor-1 (PAI-1), collectively exacerbating endothelial dysfunction, enhancing thrombogenicity, and promoting vascular smooth muscle proliferation (37). Furthermore, the NF-κB signaling pathway is activated in TAO, inducing inflammatory mediators such as TNF-α, IL-1β, IL-6, IL-8, and matrix metalloproteinases (MMPs), which contribute to endothelial injury and atherosclerotic plaque formation (38). These factors stimulate ROS production and adhesion molecule expression, reinforcing monocyte recruitment, apoptosis, and sustained inflammation. Under normal conditions, eNOS-derived NO preserves microvascular tone; however, during inflammation, inducible nitric oxide synthase (iNOS) becomes upregulated, producing excessive NO. This reacts with ROS to form peroxynitrite and other radicals, promoting oxidative damage and thrombosis. iNOS rapidly reacts with superoxide anions to generate peroxynitrite (ONOO^-^), a potent oxidant capable of inducing lipid peroxidation, protein nitration, and DNA damage within endothelial cells (39, 40). Such oxidative and nitrosative stress not only exacerbates endothelial dysfunction but also promotes a prothrombotic vascular environment, thereby directly contributing to luminal narrowing and vascular occlusion (41, 42). Endothelial cells are thus likely the primary sites of early inflammatory injury in TAO. Genetic studies reveal that the protective eNOS is significantly reduced in TAO patients, implicating the T polymorphism in disease susceptibility (5, 43). In contrast, elevated iNOS expression has been detected in endothelial cells adjacent to occluded arterial segments in TAO specimens and replicated in rat TAO models (2, 44, 45). These findings underscore the pivotal role of endothelial dysfunction, driven by altered NO signaling and inflammation, in TAO pathogenesis.

### 2.5 Endothelial activation mediated by endoplasmic reticulum stress and mitochondrial dysfunction

Recent evidence highlights endoplasmic reticulum stress (ERS) as a pivotal driver of inflammation and apoptosis through the IRE1/ASK1/JNK cascade, which also primes NLRP3 inflammasome activation (46, 47). In endothelial cells, oxidized LDL (ox-LDL) triggers ERS and ASK1 upregulation, eliciting ROS overproduction, apoptosis, inflammatory signaling, and impaired proliferation (48). Vascular remodeling in cardiovascular disease originates from endothelial dysfunction, characterized by endothelial activation, smooth muscle cell apoptosis, and extracellular matrix degradation (49). Mitochondrial dysfunction exacerbates these processes by perturbing ROS homeostasis, calcium signaling, and bioenergetics (50, 51). Under simulated microgravity, ERS-induced calcium flux promotes mitochondrial depolarization, fragmentation, and p62/SQSTM1-mediated mitophagy, diminishing endothelial barrier integrity and NLRP3 activation (52). In TAO rat models, severe ER fragmentation and mitochondrial damage in femoral arteries suggest therapeutic potential in targeting ERS–mitochondrial interplay (53). Importantly, ERS-driven mitochondrial dysfunction serves as an upstream trigger for NLRP3 inflammasome activation, resulting in the maturation and release of IL-1β and IL-18, which amplify endothelial injury and leukocyte recruitment (54–58). This proinflammatory milieu fosters platelet activation, tissue factor expression, and fibrin deposition, thereby establishing a mechanistic link between the ERS–mitochondria–NLRP3 axis and inflammatory thrombosis in TAO (59).

### 2.6 Copper and iron metabolism regulates endothelial cell activation

Copper is an indispensable trace element for systemic homeostasis. Deficiency impairs growth, causes neurological dysfunction, and downregulates adhesion molecules such as ICAM-1 and VCAM-1, thereby weakening leukocyte–endothelium interactions. In contrast, copper overload promotes endothelial activation via ICAM-1 upregulation, reversible with copper chelation (60). Excess intracellular copper drives redox cycling, elevating ROS that activate NF-κB and p38/MAPK signaling, thereby inducing ICAM-1, VCAM-1, and E-selectin expression and fostering a pro-adhesive, prothrombotic phenotype (61–65). Copper activates oxidative stress pathways and the p38 mitogen-activated protein kinase (p38/MAPK) signaling cascade, leading to endothelial cell activation, DNA damage, and cell death in human umbilical vein endothelial cells (HUVECs). Copper chelation attenuates these effects, offering potential therapeutic benefit (66). Given the elevated serum copper in TAO patients, maintaining copper homeostasis is essential to limit endothelial activation and disease progression. Ferritin, composed of light and heavy chains, stores intracellular iron; its autophagic degradation increases labile iron, fueling Fenton-driven ROS production and oxidative injury. Labile iron and heme further activate endothelial and immune cells, enhancing leukocyte and erythrocyte adhesion and precipitating vasculitis and thrombosis (67). Zinc oxide nanoparticles induce ferritinophagy in endothelial cells, elevating iron, lipid peroxidation, and dysfunction in a dose- and time-dependent manner—effects reversed by iron chelators (68). Thus, targeting iron metabolism to modulate endothelial activation represents a promising strategy for mitigating vascular inflammation and thrombosis in TAO.

### 2.7 Glycolytic regulation of endothelial activation

Endothelial activation or injury enhances vascular permeability and leukocyte adhesion, thereby promoting thrombosis and accelerating disease progression. Owing to their low mitochondrial content, endothelial cells derive 85% of ATP from glycolysis—60% sustaining homeostasis and 40% supporting proliferation (69). Even at rest, they display high glycolytic flux, which is further elevated during migration or proliferation, generating intermediates that regulate survival and function. Dysregulated glycolysis is a central driver of endothelial dysfunction and hyperproliferation (70). Loss of the glycolytic activator PFKFB3 impairs vessel formation by limiting proliferation, filopodia/lamellipodia formation, and directional migration, partly via compartmentalization with F-actin in motile protrusions (71–73). In TAO, chronic ischemia and hypoxia in distal arteries from vascular occlusion (74, 75). The local mismatch between oxygen supply and demand caused by vascular inflammation may trigger metabolic reprogramming via enhanced glycolysis (76). Targeting glycolytic pathways to restore metabolic homeostasis offers a potential strategy to ameliorate endothelial injury and vascular dysfunction in TAO (**Table 1**).

**Table 1:** Mechanisms of endothelial cell activation in thromboangiitis obliterans (TAO).

Mechanism	Molecular Factor	Pathway	Function	Therapeutic Targets
Immune Dysregulation	TNF-α, IL-6, IL-17, Autoantibodies (AECAs), CD4^+^ T cells	Immune complex formation, Activation of adhesion molecules (ICAM-1, VCAM-1), Recruitment of neutrophils, monocytes	Endothelial cell activation, Chronic inflammation, Tissue damage	Targeting pro-inflammatory cytokines (TNF-α, IL-6), Immunosuppressive therapies (corticosteroids, immunoadsorption)
Oxidative Stress	ROS, Nrf2, Cu²^+^, Fe²^+^	ROS generation, Impairment of antioxidant response (Nrf2), Endothelial dysfunction	Increased oxidative damage, Activation of NF-κB pathway, Increased thrombotic potential	Antioxidants (N-acetylcysteine), Nrf2 activators
eNOS/iNOS Dysregulation	eNOS, iNOS, NO, ROS	Decreased NO bioavailability, Increased peroxynitrite (ONOO^-^) production	Impaired vasodilation, Increased platelet aggregation, Endothelial dysfunction	NOS inhibitors (L-NAME), NO donors (Sodium Nitroprusside)
Mitochondrial Dysfunction	ROS, Calcium flux, Mitochondrial DNA, ER stress (CHOP, ATF4)	Impaired mitochondrial biogenesis, Increased ROS production, Activation of mitochondrial apoptosis pathways	Energy deficiency in ECs, Endothelial cell apoptosis, Vascular remodeling	Mitochondrial-targeted antioxidants, ER stress modulators (ISRIB)
Copper and Iron Imbalance	Cu²^+^, Fe²^+^, Ferritin, Ceruloplasmin, Superoxide dismutase	Disruption of copper and iron homeostasis, Activation of oxidative stress pathways, Iron-driven ROS production	Increased endothelial activation, Thrombosis, Vascular dysfunction	Copper chelation (Trientine), Iron chelators (Deferoxamine)
Glycolysis Reprogramming	GLUT1, PFKFB3, Hexokinase, JAK/STAT, NF-κB	Increased glycolytic activity in ECs, Altered metabolic pathways in response to hypoxia	Endothelial cell proliferation and dysfunction, Increased thrombotic risk	Glycolysis inhibitors (2-DG), JAK/STAT inhibitors
Inflammatory Signaling	NF-κB, MAPKs, JAK/STAT, IL-6, IL-1β	Activation of transcription factors (NF-κB, AP-1), Upregulation of adhesion molecules, Pro-inflammatory cytokine release	Chronic vascular inflammation, Platelet aggregation, Thrombosis	NF-κB inhibitors (BMS-345541), JAK inhibitors (Tofacitinib)

### 2.8 Inflammation-associated signaling pathways regulate endothelial cell activation

**Figure 1:**
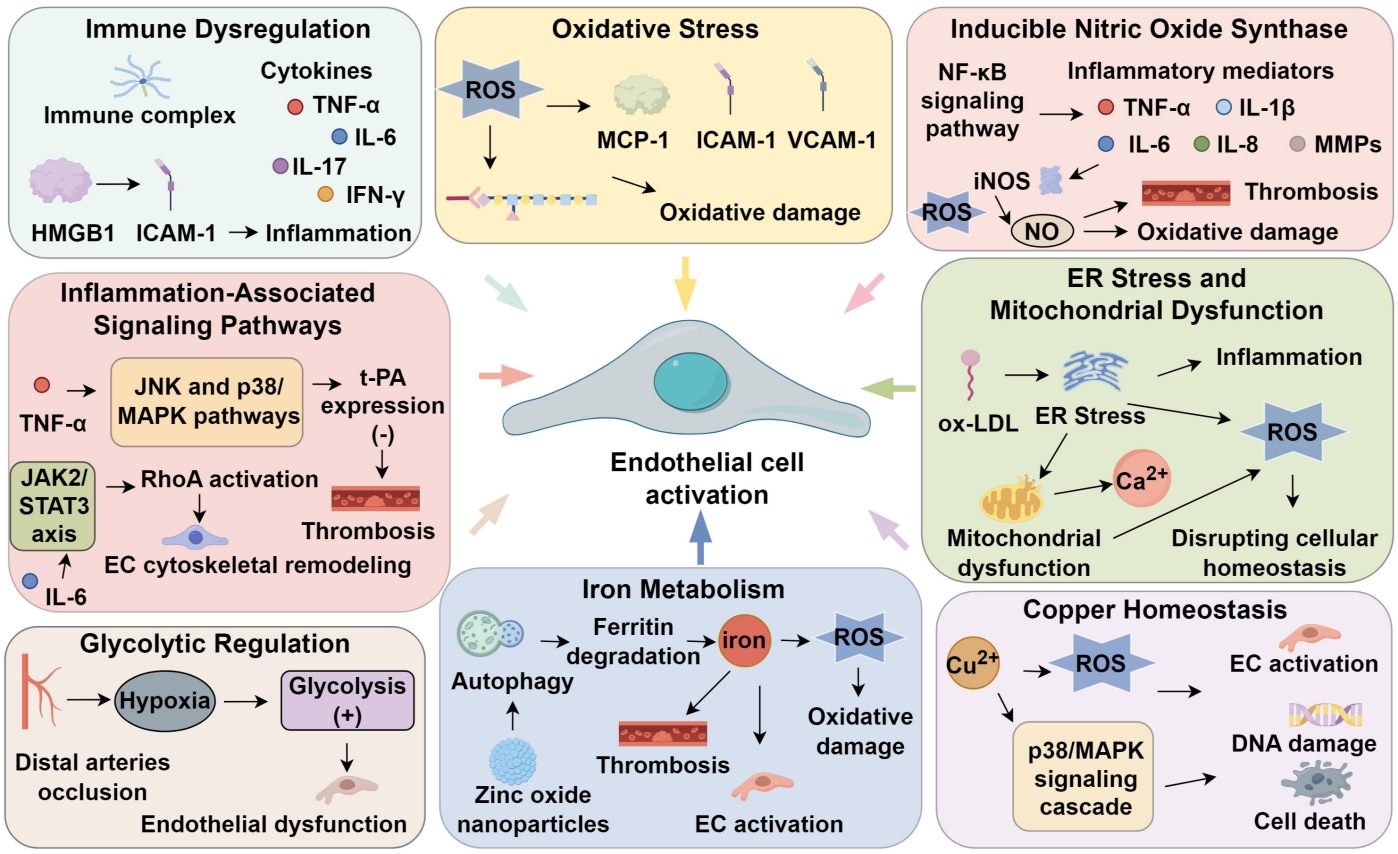
Endothelial activation in thromboangiitis olterans.

Endothelial dysfunction is a hallmark of TAO, driving inflammation, thrombosis, and vascular remodeling. In thrombohemorrhagic vasculitis, spleen tyrosine kinase (Syk) activation within vascular walls triggers neutrophil elastase release, promoting hemorrhage, fibrin deposition, and thrombosis (77). Beyond adaptive immunity, Syk orchestrates cell adhesion, innate immune activation, osteoclast differentiation, platelet aggregation, and vascular development (78), with Syk–MAPK signaling being pivotal for pro-inflammatory gene expression and NLRP3 inflammasome activation (79). Licochalcone A attenuates endothelial activation by inhibiting Syk phosphorylation, suppressing p38/JNK signaling, and reducing NLRP3-driven IL-1β and IL-18 secretion (61). Endothelial cells exhibit both antithrombotic and prothrombotic properties. Under homeostasis, they prevent thrombosis via prostacyclin, tissue plasminogen activator (t-PA), and tissue factor pathway inhibitor. However, injury shifts the balance toward thrombogenesis, enhancing platelet aggregation and thrombus formation. t-PA promotes fibrinolysis through plasmin activation, while its inhibitor, plasminogen activator inhibitor-1 (PAI-1), facilitates thrombosis by impeding fibrinolysis and enhancing platelet adhesion (80). A reduced t-PA/PAI-1 ratio critically increases thrombotic risk (81). The NF-κB pathway governs endothelial-platelet-cytokine crosstalk, disrupting coagulation-fibrinolysis homeostasis. Activation of NF-κB and AP-1 reflects upstream signaling via JNK and p38/MAPK pathways. TNF-α suppresses t-PA expression via these axes, further impairing fibrinolysis (82). Compounds such as shikonin and salvianolic acid B counteract endothelial injury and thrombosis by modulating the NF-κB/JNK/p38/MAPK pathway (83). Additionally, the JAK2/STAT3 axis regulates endothelial cytoskeletal remodeling via downstream activation of RhoA, a GTPase involved in adhesion and motility. IL-6–driven STAT3 activation enhances RhoA/ROCK signaling, altering actin dynamics and focal adhesion (84). In TAO, IL-6/STAT3 signaling disrupts endothelial homeostasis by modulating adhesion molecules and cytoskeletal structure (20). In conclusion, persistent endothelial activation in TAO is mechanistically linked to immune signaling, inflammation, and thrombosis. Disease severity correlates with the extent of endothelial injury, implicating it as a potential initiating factor. Targeting these signaling cascades may restore endothelial integrity and offer novel therapeutic strategies for TAO (**Figure 1**).

## 3 Advances in the treatment of TAO

Common oral agents for TAO include aspirin, tolazoline, batroxobin, and prostaglandin analogs (85). Argatroban, a direct thrombin inhibitor targeting its catalytic site, exhibits greater efficacy when combined with prostaglandin E1 (PGE1) than PGE1 alone (86), effectively ameliorating hypercoagulability and improving therapeutic outcomes. Prostaglandin analogs, which enhance peripheral circulation, are widely employed in ischemic limb disorders. Comparative analyses demonstrate their superiority over open lumbar sympathectomy in achieving complete ulcer healing, relieving rest pain, and reducing major amputation rates (87, 88).

Lumbar sympathectomy and endarterectomy remain cornerstone surgical interventions for TAO, targeting sympathetic denervation of the lower limbs to relieve vasospasm and enhance distal vasodilation and perfusion (89). Percutaneous transluminal angioplasty (PTA), including balloon dilation, stent placement, thrombolysis, and thrombectomy, restores vessel patency by resolving occlusive lesions (90), with balloon angioplasty being the most widely applied and demonstrating substantial efficacy (91). Clinical evidence shows that PTA significantly promotes ulcer healing, alleviates pain, and preserves limbs (92). In one series, distal perfusion improved and the limb salvage rate reached 92% (93), while another reported 100% clinical efficacy with marked gains in iliac artery flow and ankle–brachial index (64). For femoropopliteal TAO, excimer laser ablation combined with drug-coated balloon angioplasty achieved 92.0% 12-month patency (94). Although short-term outcomes are encouraging, long-term durability and complication profiles remain to be clarified. Tibial transverse bone transport (TTBT), which promotes microvascular regeneration via longitudinal tibial traction, has been shown to restore lower-limb microcirculation, reduce ischemic pain, improve hemorheology, and enhance wound healing and ankle–brachial index (95).

Stem cell transplantation offers a promising option for patients unresponsive to revascularization or conservative therapy, improving symptoms and lowering amputation rates (96, 97). Comparative studies of peripheral blood mononuclear cells (PBMC) and purified CD34^+^ cells (PCC) revealed satisfactory long-term outcomes, with PBMC yielding faster symptom relief and PCC causing less injection site discomfort (98, 99). Stem cell therapy attenuates endothelial activation by restoring nitric oxide synthase activity, reducing oxidative stress, and downregulating ICAM-1/VCAM-1 expression, thereby improving endothelial barrier integrity (100–103). Additionally, transplanted stem cells secrete pro-angiogenic factors such as VEGF, FGF, and angiopoietins, which stimulate neovascularization in ischemic tissues (104–106). These paracrine effects also modulate immune responses by shifting the Th1/Th17-driven pro-inflammatory milieu toward an anti-inflammatory Treg-dominant profile, potentially mitigating the autoimmune-mediated endothelial injury (107, 108). Endovascular radiofrequency ablation (ERA), which ablates sympathetic ganglia to reduce vasoconstriction, is minimally invasive and effective for ischemic pain relief (109), though current evidence suggests a higher complication rate compared with cell-based or intra-arterial approaches, warranting larger studies.

## 4 Conclusion

TAO is a multifactorial vasculopathy in which sustained endothelial activation represents the central pathogenic driver. This state is propagated by autoimmune processes such as anti-endothelial cell antibodies and Th17-mediated inflammation, coupled with oxidative stress from Nrf2/ROS disequilibrium, nitric oxide synthase dysregulation, and copper/iron metabolic imbalance. These insults converge to induce a pro-adhesive, pro-thrombotic endothelial phenotype that fosters leukocyte recruitment, vascular inflammation, and luminal occlusion. Endoplasmic reticulum–mitochondrial crosstalk amplifies NLRP3 inflammasome activation, while glycolytic metabolic reprogramming sustains endothelial activation under hypoxia, culminating in progressive microvascular failure and tissue ischemia.

Current medical, surgical, and endovascular interventions from prostaglandin analogues to percutaneous transluminal angioplasty offer symptomatic relief and delay amputation but do not address upstream endothelial dysfunction. Emerging strategies including stem cell transplantation, targeted immunomodulation, and metabolic correction aim to restore endothelial homeostasis by enhancing nitric oxide bioavailability, reducing oxidative stress, and promoting a Treg-skewed immune profile. Yet, their durability, safety, and patient-specific efficacy remain undefined. Future work should focus on precision medicine, integrating genetic, epigenetic, and biomarker-guided stratification to enable mechanism-based therapy, supported by large-scale longitudinal trials to transition TAO care from symptom palliation to durable disease modification.
